# Protease-activated receptors (PARs): mechanisms of action and potential therapeutic modulators in PAR-driven inflammatory diseases

**DOI:** 10.1186/s12959-019-0194-8

**Published:** 2019-03-29

**Authors:** Dorothea M. Heuberger, Reto A. Schuepbach

**Affiliations:** 1 Institute of Intensive Care Medicine, University Hospital Zurich, University of Zurich, Zurich, Switzerland; 2 Surgical Research Division, University Hospital Zurich, University of Zurich, Zurich, Switzerland

## Abstract

Inflammatory diseases have become increasingly prevalent with industrialization. To address this, numerous anti-inflammatory agents and molecular targets have been considered in clinical trials. Among molecular targets, protease-activated receptors (PARs) are abundantly recognized for their roles in the development of chronic inflammatory diseases. In particular, several inflammatory effects are directly mediated by the sensing of proteolytic activity by PARs.

PARs belong to the seven transmembrane domain G protein-coupled receptor family, but are unique in their lack of physiologically soluble ligands. In contrast with classical receptors, PARs are activated by N-terminal proteolytic cleavage. Upon removal of specific N-terminal peptides, the resulting N-termini serve as tethered activation ligands that interact with the extracellular loop 2 domain and initiate receptor signaling. In the classical pathway, activated receptors mediate signaling by recruiting G proteins. However, activation of PARs alternatively lead to the transactivation of and signaling through receptors such as co-localized PARs, ion channels, and toll-like receptors.

In this review we consider PARs and their modulators as potential therapeutic agents, and summarize the current understanding of PAR functions from clinical and in vitro studies of PAR-related inflammation.

## Introduction

**Fig. 1:**
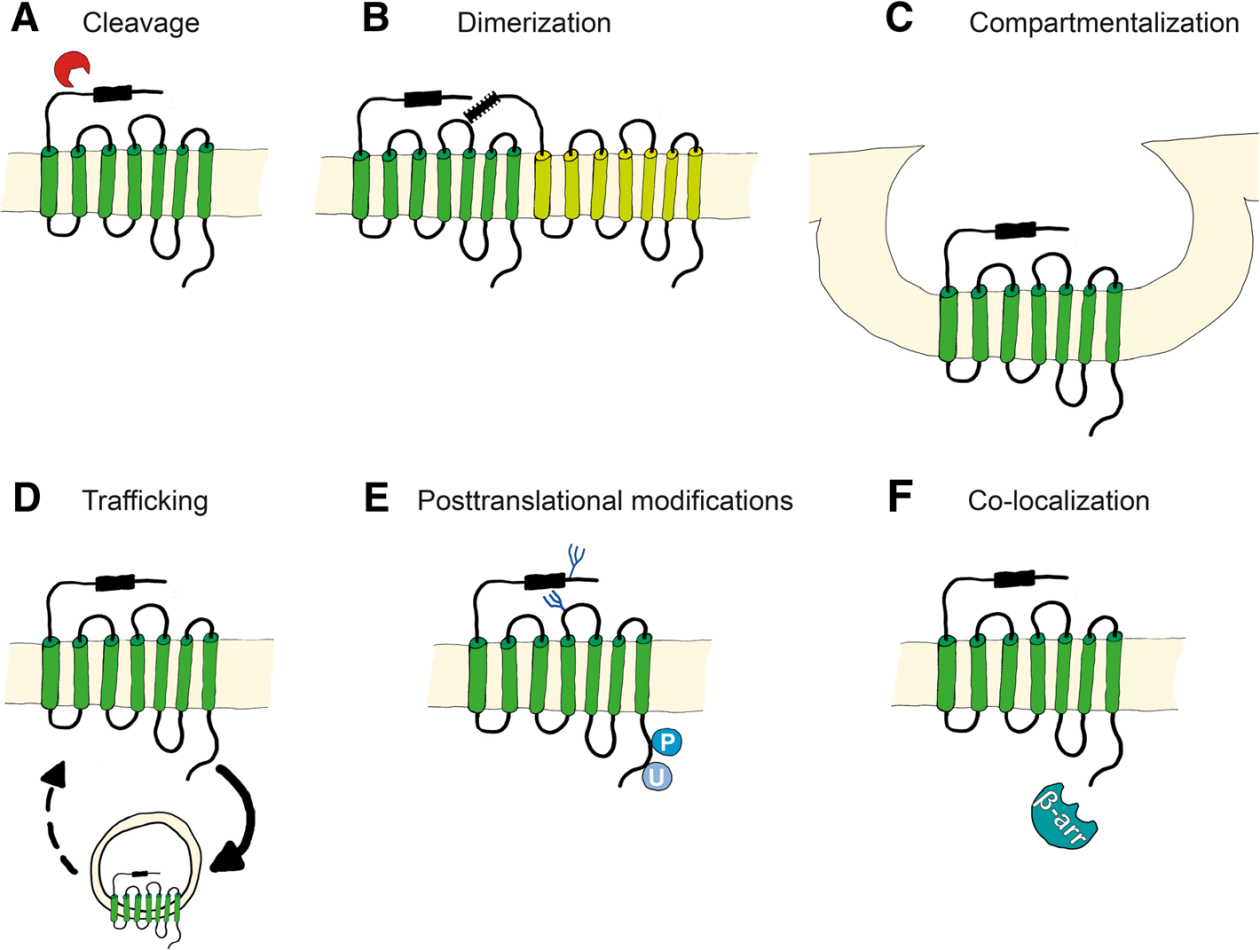
Mechanisms of PAR activation. PAR activation is regulated by **a** direct proteolytic cleavage at the N-terminus, **b** homo- or heterodimerization with other PARs and transactivation through the cleaved tethered ligand, **c** compartmentalization on the cell surface, **d** degradation or recycling by endosomal trafficking, **e** posttranslational modifications such as glycosylation, phosphorylation, and ubiquitination, and **f** co-localization with other receptors and cofactors

The four mammalian members of the protease-activated receptor (PAR) family PAR1, PAR2, PAR3, and PAR4 are encoded by the genes F2R [1], F2RL1 [2], F2RL2 [3], and F2RL3 [4], respectively. Human PAR1 was discovered in 1991 as a key thrombin receptor on platelets [5, 6]. Although human and mouse PAR2 genes are homologous to PAR1 genes, PAR2 is not responsive to thrombin [2, 7, 8]. Unexpected responses of platelets to thrombin in PAR1 knockout mice lead to the discovery of the thrombin receptors PAR3 and PAR4 [4, 9, 10]. PAR regulation varies between species and tissues, with differing expression levels, protease cleaving activities, dimerization with other receptors, compartimentalization, trafficking, posttranslational modifications, and co-localization with co-receptors, as shown in Fig. 1.

Studies of PAR activation under physiological conditions are crucial for the understanding of the pathophysiological roles of PARs, such as those in inflammatory disorders.

## Cleavage and activation of PARs and signal transduction

PARs are specifically cleaved and irreversibly activated by various endogenous proteases, and by exogenous proteases from bacteria, plants, fungi, and insects. Proteases, soluble or cell membrane associated (bound to co-receptors or specific membrane compartments), cleave specific N-terminal peptides of PARs, resulting in exposure of new N-terminal peptides that serve as tethered activation ligands, which bind a conserved region on extracellular loop 2 (ECL2) [5, 11]. This interaction initiates conformational changes and alters affinity for intracellular G proteins [12]. Various N-terminal cleavage sites have been described, and these have various active conformations with specific G protein preferences. Multiple cleavage site-specific cellular responses are generally referred to as biased signaling, and the ensuing models describe how distinct proteases with distinct cleavage sites induce protease-specific responses via the same PAR [13, 14].

In contrast with PAR-activating proteases, other proteases cleave PARs at cleavage sites that are not related to signaling. Under these conditions, shedding of the PAR1 terminus, which removes the thrombin activation site, was first recognized as a mechanism for rendering platelets irresponsive to thrombin [15]. These truncated PARs can no longer be proteolyticaly activated, but remain activated by ligands from adjacent PARs [16]. Alternatively, truncated PARs bind soluble peptides with affinity for ECL2 by mimicking the tethered ligand. Both mechanisms result in receptor activation [17, 18]. Multiple ECL2-binding agonist peptides have been described and shown to induce signaling from truncated and uncleaved PARs (see agonist peptides in Tables 5, 6, 7).

### PAR activation by proteolytical cleavage

**Fig. 2:**
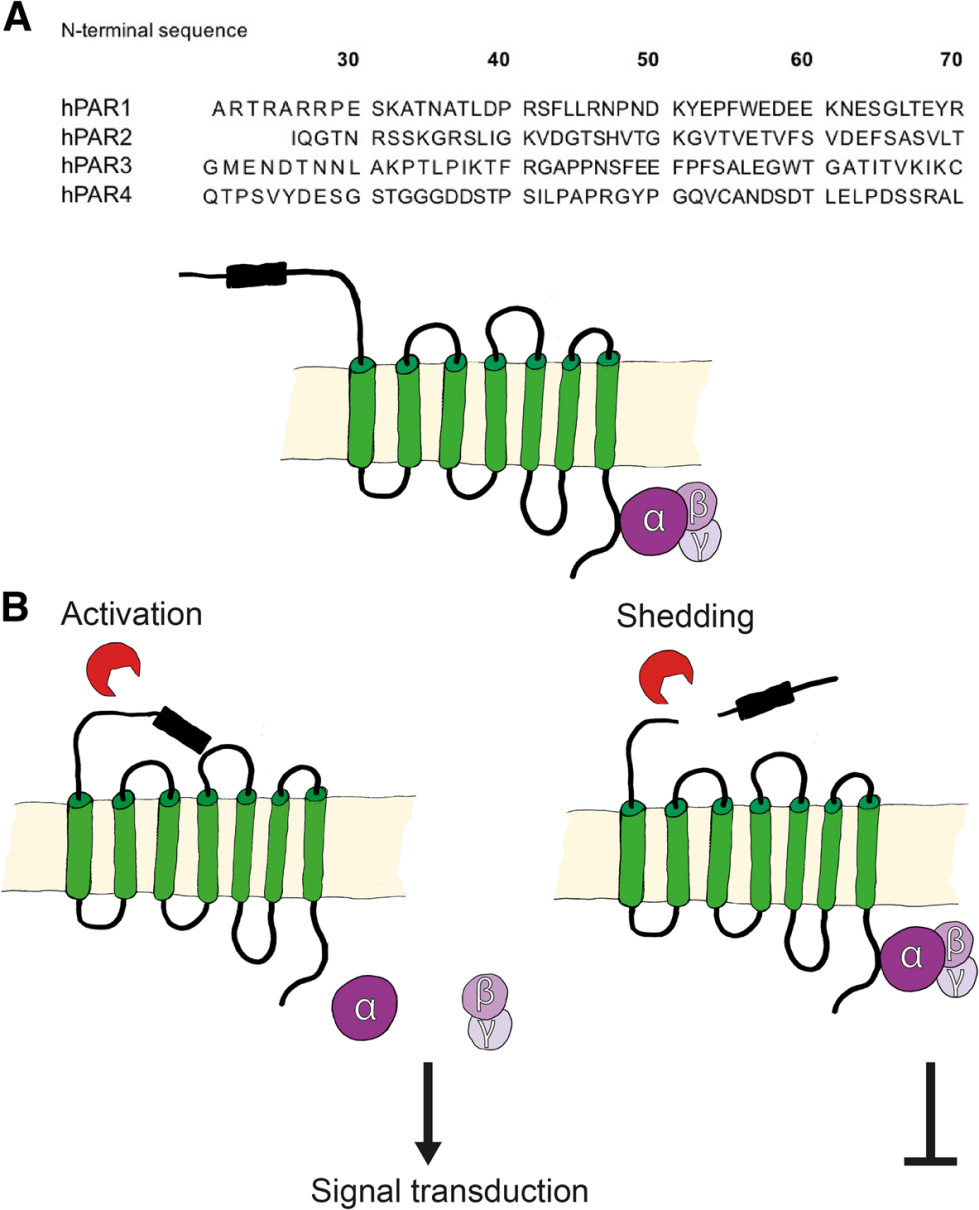
Proteolytic PAR cleavage. **a** N-terminal sequences of human PARs (PAR1–4) containing potential cleavage sites. **b** Proteolytic cleavage of PARs by soluble exogenous proteases exposes new N-terminal sequences that serve as tethered ligands for G protein dependent activation of receptors. Alternatively, proteolytic cleavage at other sites destroys the function of the receptor to prevent intracellular signal transduction

PAR-cleaving proteases are a focus of many current studies. Whereas some PAR-cleaving proteases produce N-terminal components with regulatory roles, others render the receptors irresponsive to further protease exposure as shown in Fig. 2 and summarized in Tables 1, 2, 3 and 4. Important proteases are discussed below.

**Table 1:** PAR1 cleaving proteases

	Protease	Major cleavage site	Additional cleavage sites
Mammalian proteases	Thrombin	R^41^S^42^	
	aPC	R^46^N^47^	R^41^S^42^
	FVIIa	unknown	
	FXa	R^41^S^42^	
	Trypsin	R^41^S^42^	
	Chymase	unknown	
	MMP-1	D^39^P^40^, L^44^L^45^, F^87^I^88^	N^47^P^48^, R^70^L^71^,K^82^Q^83^
	MMP-2	L^38^D^39^	
	MMP-3,-8,-9	R^41^S^42^	
	MMP-12	unknown	
	MMP-13	S^42^F^43^	L^38^T^39^, mouse
	Cathepsin G	R^41^S^42^, F^55^W^56^, Y^69^R^70^	
	Neutrophil elastase	A^36^T^37^, V^72^S^73^, A^86^F^87^	
	Proteinase-3	A^36^T^37^, P^48^N^49^, V^72^S^73^, A^92^S^93^	
	Plasmin	K^32^A^33^, R^41^S^42^, R^70^ L^71^, K^76^ S^77^, K^82^ Q^83^	
	Kallikrein-4,-5,-6	unknown	
	Kallikrein-14	R^46^N^47^	
	Granzyme A,B, K	unknown	
	Calpain-1	K^32^A^33^, S^76^K^77^	
Non-mammalian proteases	PA-BJ	R^41^S^42^, R^46^N^47^	
	Thrombocytin	R^41^S^42^, R^46^N^47^	
	DerP1	unknown	
	Gingipain R	R^41^S^42^	
	SpeB	L^44^L^45^	
	LepA	unknown	
	*S.pneumoniae* proteases	unknown	
	Thermolysin	F^43^L^44^, L^44^L^45^	
	penC	R^41^S^42^

**Table 2:** PAR2 cleaving proteases

	Protease	Major cleavage site	Additional cleavage sites
Mammalian proteases	Thrombin	R^36^S^37^	
	aPC	unknown	
	FXa	R^36^S^37^	
	Trypsin	R^36^S^37^	K^34^G^35^, K^51^G^52^, K^72^L^73^
	Tryptase	R^36^S^37^	
	Chymase	G^35^R^36^	L^38^I^39^, mouse
	Matriptase	R^36^S^37^	
	Cathepsin G	F^65^S^66^	F^59^S^60^, F^64^S^65^
	Cathepsin S	G^40^K^41^	E^56^P^57^, mouse
	Neutrophil elastase	A^66^S^67^, S^67^V^68^	V^42^D^43^,V^48^T^49^,V^53^T^54^,V^58^T^59^,T^74^T^75^,V^76^F^77^
	Proteinase-3	D^62^E^63^	V^48^T^49^,V^55^E^56^,T^57^V^58^ V^61^D^62^,K^72^L^73^,T^74^T^75^,T^75^V^76^,V^76^F^77^
	Plasmin	R^36^S^37^	K^34^G^35^
	Testisin	unknown	
	Kallikrein-4,	unknown	
	Kallikrein-5,-6,-14	R^36^S^37^	
	Calpain-2	unknown	
Non-mammalian proteases	Der-P1,-P2,-P3,-P9	unknown	
	Cockroach E1-E3	R^36^S^37^	
	Gingipain R	unknown	
	LepA	unknown	
	EPa	S^37^L^38^	S^38^L^39^, rat
	*S.pneumoniae* proteases	unknown	
	Thermolysin	unknown	
	Serralysin	unknown	
	*P.acnes* proteases	unknown	
	aPA	unknown	
	Bromelain	unknown	
	Ficin	unknown	
	Papain	unknown	
	penC	R^36^S^37^

**Table 3:** PAR3 cleaving proteases

	Protease	Major cleavage site	Additional cleavage sites
Mammalian proteases	Thrombin	K^38^T^39^	mouse PAR3 at K^37^S^38^
	aPC	R^41^G^42^	
	FXa	R^41^G^42^	
	Trypsin	unknown

**Table 4:** PAR4 cleaving proteases

	Protease	Major cleavage site	Additional cleavage sites
Mammalian proteases	Thrombin	R^47^G^48^	
	Trypsin	R^47^G^48^	
	Cathepsin G	R^47^G^48^	
	Kallikrein-14	unknown	
Non-mammalian proteases	PA-BJ	R^47^G^48^	
	Thrombocytin	R^47^G^48^	
	Der-P3	unknown	
	Gingipain R	R^47^G^48^	
	LepA	unknown	
	*S.pneumoniae* proteases	unknown	
	Bromelain	unknown	
	Ficin	unknown	
	Papain	unknown	

#### Mammalian proteases

##### Serine proteases

Thrombin, the key protease of coagulation, is generated by proteolytic cleavage of zymogen prothrombin. Although thrombin production predominantly occurs on platelets and subendothelial vascular walls, extravascular thrombin has been detected in synovial fluid [19] and around tumors [20]. Thrombin has long been known to activate platelets, and the discovery of PAR1 initiated research into the underlying molecular mechanisms. PAR1 contains a hirudin-like domain, which has a high affinity thrombin binding site and recruits thrombin via exosite I. This interaction enables thrombin to specifically and efficiently activate PAR1 [6]. Similarly, PAR3 contains a hirudin-like thrombin recruitment site, which results in cleavage [9, 21]. In other studies, mouse PAR3 maintained thrombin recruitment activity but lost its receptor function, as discussed above [22–24]. Thrombin also cleaves and activates PAR4, which, in contrast with PAR1, lacks a hirudin-like domain. Thus, higher concentrations of thrombin activate PAR4 and initiate intracellular signaling [10]. PAR2 is considered the only PAR that resists cleavage or activation by thrombin [4, 25], although emerging evidence suggests that at very high concentrations (100–500 nM), thrombin may directly cleave and activate PAR2 [26, 27].

In contrast with thrombin, the anticoagulant protease activated protein C (aPC) binds to the co-receptor endothelial protein C receptor (EPCR) to promote the cleavage and activation of co-localized PAR1 [28, 29] and induce anti-apoptotic and protective effects on endothelial barrier permeability [29–33]. Compartmentalization of PAR1 and co-localization with EPCR in calveolae is crucial for efficient cleavage by aPC [13]. Moreover, aPC cleaves PAR3 in humans and mice [21, 34, 35] and acts as a PAR3 shedding protease that prevents thrombin-induced barrier disruption [21]. However, the dependency of aPC cleavage of PAR3 on EPCR remains controversial [21, 35]. Similar to aPC, coagulation factor Xa binds EPCR and mediates proteolytic activation of PAR1 and PAR3 [21, 28, 36–39]. In addition, EPCR-bound factor Xa reportedly cleaves PAR2 and initiates inflammatory signaling [40]. PAR2 was also shown to be activated by tissue factor (TF)-bound coagulation factor VIIa [40–42]. Yet recent studies suggest that the TF-VIIa complex does not directly activate PAR2, and rather activates matriptase, which cleaves and activates PAR2 [42–44]. Anti-inflammatory signaling was also previously related to PAR1 cleavage by EPCR-bound VIIa [45, 46]. Taken together, these studies indicate that TF-Xa–VIIa complexes activate PAR1 and PAR2 [47].

Trypsins are PAR-activating proteases with roles as major digestive enzymes in the duodenum [48]. Trypsin is also secreted by epithelial cells, nervous system cells [49], and tumor cells [50, 51]. Trypsins may also be involved in cell growth and coagulation, as suggested by secretion from human vascular endothelial cells [52]. Trypsin cleaves human PAR1 and PAR4 at putative protease cleavage sites, and thereby prevents thrombin signaling in endothelial cells and platelets [4, 53]. Trypsin is the major PAR2 cleaving protease that initiates inflammatory signaling [2, 7].

Tryptase is the main protease of mast cells, and activates PAR2 by proteolytic cleavage to induce calcium signaling and proliferation [54–57]. The source tissue of tryptase reportedly plays an important role in the cleavage and induction of tryptase-activated PAR signaling, reflecting differences in posttranslational modifications, such as glycosylation and sialic acid modifications [54, 58]. Tryptase induces calcium signaling via PAR1 when PAR2 is co-expressed, but cannot activate human platelets, suggesting that tryptase does not directly cleave PAR1 [54–57]. Chymase is a mast cell serine protease that also cleaves PAR1 in human keratinocytes and fibroblasts, and thus prevents thrombin sensitivity [59]. Moreover, the epithelial serine protease matriptase cleaves and initiates inflammatory responses in human and mouse keratinocytes and in *Xenopus* oocytes overexpressing human PAR2 [44, 60–63].

PARs have been identified as substrates of kallikreins, which are serine proteases that have been related to various inflammatory and tumorigenic processes [64]. Kallikrein-4 increases intracellular calcium levels via PAR1 and PAR2, but activates PAR1 most efficiently [65]. Kallikrein-14 induces calcium signaling via PAR1, PAR2, and PAR4, but can also shed PAR1 to prevent signaling. Rat platelets are activated by kallikrein-14 via the proteolytic cleavage of PAR4, but are not activated by kallikrein-5 and kallikrein-6 [66]. Instead, neurotoxic effects of kallikrein-6 were inhibited by blocking PAR1 and PAR2, indicating a direct proteolytic role in PAR activation [67].

Neutrophils are mobilized to sites of inflammation and infection, where they modulate inflammatory signaling, in part by secreting PAR-cleaving proteases. The neutrophil serine protease cathepsin G prevents thrombin-induced effects by cleaving PAR1 into non-functional parts [68, 69]. In contrast, cathepsin G reportedly induced chemoattractant signaling via PAR1, further supporting the role of cathepsin G in PAR1 activation [70]. Another unexpected observation of cathepsin G was that cleavage sites differ between recombinant and native human PAR2 [26, 71, 72]. These discrepancies may reflect the influence of cell types and posttranslational modifications on PAR cleavage. Studies in mice and humans show that platelet activation by cathepsin G is dependent on PAR3 and PAR4 [71, 73, 74]. Cathepsin G also cleaves and activates PAR4 on endothelial cells [75]. The neutrophil proteases elastase and proteinase-3 cleave recombinant PAR1 and PAR2 at various sites [26, 72]. Recently, rat elastase was shown to cleave and activate PAR1, although sequences of rat and human PAR1 have low homology [76]. In contradiction with neutrophil proteases that prevent PAR signaling at sites of inflammation, monocytes secret the protease cathepsin S, which initiates inflammatory signaling by cleaving PAR2 [72, 77, 78]. Low concentrations of the fibrinolytic protease plasmin prevent platelet activation by cleaving PAR1, whereas high concentrations of plasmin lead to the cleavage and activation of PAR1 [79]. Plasmin also cleaves PAR2 and prevents subsequent activation by trypsin [26, 80].

The serine proteases granzyme A and granzyme B induce intracellular signaling pathways that lead to neuronal death via PAR1 [81, 82]. Recently, granzyme K was also shown to activate PAR1 and promote inflammatory endothelial signaling [83, 84]. Few studies show activation of PAR1 by proteases of the granzyme family, and the details of this interaction remain poorly characterized.

##### Cysteine proteases

Calpain-1 is a calcium-dependent cysteine protease that has been associated with inflammatory disorders, and initiates calcium signaling pathways by activating PAR1 [26]. At very high concentrations, calpain-2 was also shown to cleave PAR2, and the authors suggested that this cleavage event inactivated PAR2 [26]. Recently, calpain-1 was shown to be induced by thrombin-activated PAR1, and subsequently regulated the internalization of PAR1 [85].

##### Metalloproteases

Matrix metalloproteases (MMPs) are known to be involved in various inflammatory- and cancer-related conditions. MMP-1 cleaves human PAR1 and initiates platelet activation [86–89]. MMP-1 also regulates cancer cell activities depending on PAR1 availability [90]. Similarly, MMP-2 cleaves human PAR1 and enhances platelet activation [91], and MMP-3, MMP-8, and MMP-9 were shown to induce platelet activation via PAR1 [92]. Whether these three MMPs cleave PAR2 is not clear, although PAR2 activation by trypsin induced secretion of MMP-9 in human airways, suggesting that MMP-9 is a PAR2-activating protease [93]. In mice, PAR1 expression was regulated by MMP-12, and activated PAR1 increased MMP-12 secretion [94, 95]. A similar feedback loop involving MMP-12 and PAR2 has been reported in mice [96]. Moreover, MMP-13 was shown to activate PAR1 and induce intracellular signaling [87], and thrombin-induced activation of PAR1 and PAR3 was associated with increased levels of MMP-13 in human chondrocytes [24].

In addition to coagulation and inflammation, PAR activation may play roles in human germ cells, where the serine protease testisin activates PAR2 and induces calcium signaling and ERK1/2 activation. This interaction may play roles in the regulation of ovarian and testicular cancer, as suggested previously [97, 98].

#### Non-mammalian proteases

**Fig. 3:**
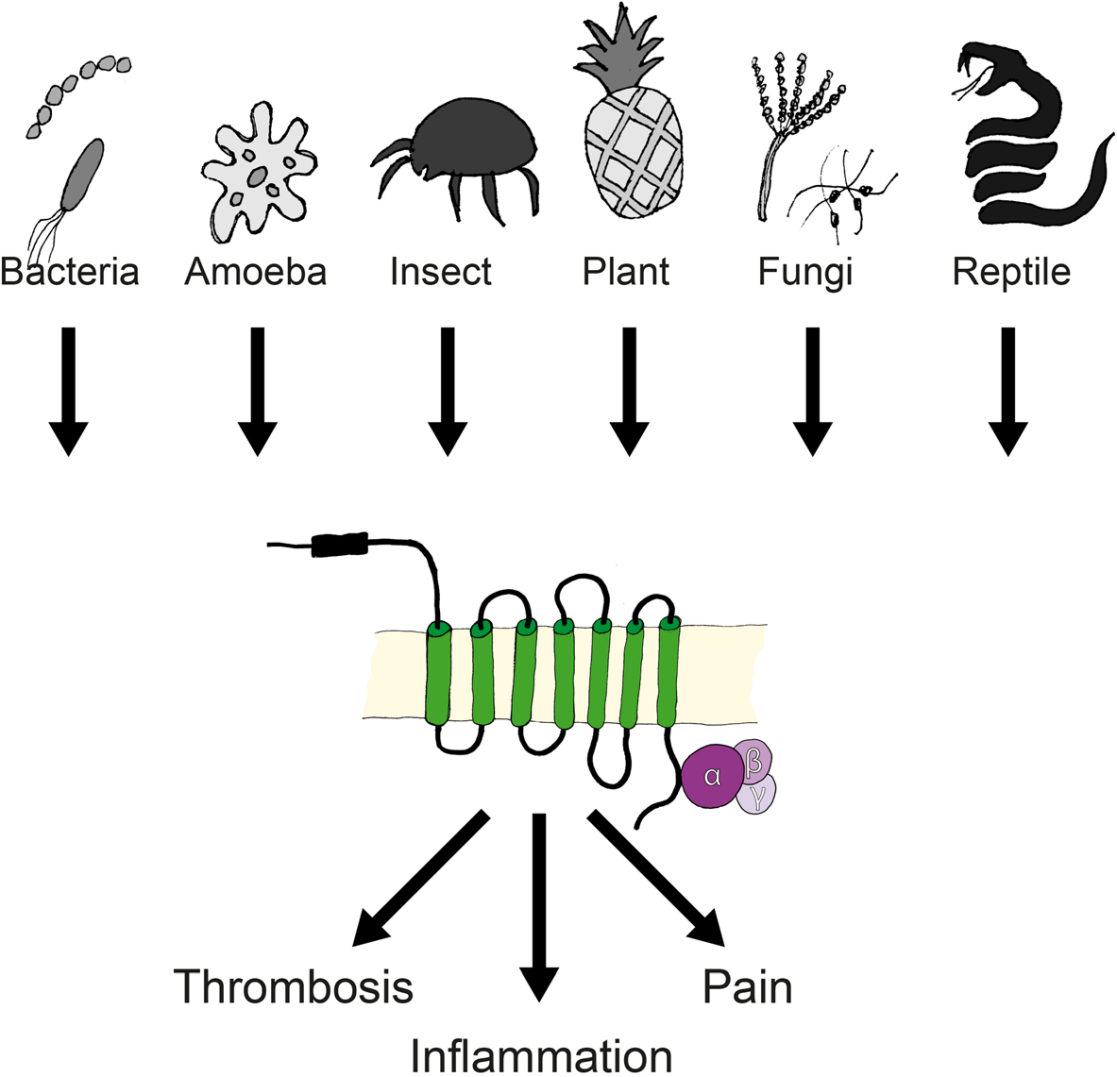
Non-mammalian exogenous proteases induce PAR-driven pathological effects. Various proteases are secreted from bacteria, amoebae, insects, plants, fungi, and snakes, and can cleave PARs and modulate signal transduction, leading to inflammation, thrombosis, or pain

Exogenous proteases from various species that modulate PAR activation are disscues in the following section and are summarized in Fig. 3.

##### Bacterial proteases

Endogenous mammalian proteases are not the only regulators of PAR activation. Indeed, both pathogenic and commensal bacteria secret various proteases that cleave PARs and act as inflammatory modulators [99]. In this section, we describe bacterial proteases that either activate PARs, and thus allow bacteria to penetrate host barriers, or inactivate PARs to prevent inflammatory signaling by the host.

The human pathogen *Pseudomonas aeruginosa* secrets two PAR-cleaving proteases with contrasting effects. The exoprotease LepA cleaves and activates PAR1, PAR2, and PAR4, and subsequently induces nuclear factor kappa B (NFκB) promoter activity [100], whereas cleavage by elastase EPa inactivates PAR2 to prevent inflammation in lungs [101].

The streptococcal pyrogenic exotoxin B (SpeB) of Group A *Streptococcus* also inactivates PAR1 by cleaving it, and thereby renders human platelets unresponsive to thrombin [102]. In mice, proteases of *Streptococcus pneumoniae* cleaved PAR2 and facilitated the spread of the pathogen from the airways into the blood stream [103]. PAR1 has also been associated with *S. pneumonia-*mediated sepsis in mice, although direct cleavage of PAR1 was not shown [104, 105]. Pulmonary inflammation from *S. pneumoniae* infections is reduced in PAR4 knockout mice [106], further supporting this causal link.

Inflammation-associated periodontal diseases are predominantly induced by the *Porphyromonas gingivalis* cysteine protease gingipain R, which activates PAR2 [107, 108]. Subsequently, gingipain R activates PAR1 and PAR4, and thereby, human platelets [109–111]. This mechanism may also explain associations between periodontitis and cardiovascular events [112].

In addition, supernatants from *Propionibacterium acnes* cultures initiated inflammatory signaling in human keratinocytes via PAR2 [92]. The virulence of *P. acnes* was also reduced in PAR2 knockout mice [113], further suggesting that PAR2 is involved in bacterial infections.

Serralysin is a matrix metalloprotease expressed by *Serratia marcescens*, and induced inflammation in human airway cells via PAR2 in vitro [114].

Finally, *Bacillus thermoproteolyticus rokko* secretes the metalloprotease thermolysin, which cleaves and inactivates PAR1 to prevent thrombin-induced signaling in rat astrocytes [115, 116]. The in vitro effects of PAR2-cleavage by thermolysin, however, vary between cell lines [116].

##### Amoeba proteases

In acanthamoebic keratitis, PAR2 triggers inflammation following secretion of the plasminogen activator (aPA) by *Acanthamoeba* strains, leading to induction of IL-8 in human corneal epithelial cells [117].

##### Reptile proteases

Following snakebites, coagulation disorders in humans and mice occur due to the presence of venom proteases. In *Proatheris superciliaris* bites, venom proteases activate platelets by activating PAR1 and PAR4 [118]. *Bothrops atrox* and *B. jararaca* are snake species of the family viperidae. These snakes secrete the serine proteases PA-BJ and thrombocytin, which activate human platelets via PAR1 and PAR4 [119].

##### Insect proteases

Several cysteine and serine proteases from insects induce inflammation-associated diseases such as asthma. For example, dust mite allergens contain the serine proteases DerP2, DerP3, and DerP9 [120] and the cysteine protease DerP1. DerP1 induces PAR2-dependent signaling, whereas thrombin-induced PAR1-signaling is prevented by these proteases in human epithelial cells [121]. DerP3 was also recently shown to activate PAR4, and this process was associated with allergies to dust mites [122].

Similar to proteases from house dust mites, three serine proteases (E1–E3) from cockroach extracts activate PAR2 and induce inflammatory signaling in mice and humans [123–125].

##### Fungal proteases

Pen C is a serine protease from *Penicillium citrinum* that induces IL-8 in human airway cells by activating PAR1 and PAR2 [126]. Proteases from *Aspergillus fumigatus* have also been shown to prevent PAR2-dependent inflammation [127]. Moreover, serine proteases from *Alternaria alternate* induced calcium signaling in human bronchial cells and induced inflammation in mice by secreting IL-33 following PAR2 activation [128–130].

##### Plant proteases

Bromelain is a mixture of cysteine proteases that is extracted from pineapple which is used as a PAR-independent anti-inflammatory agent [131]. Bromelain cleaves PAR2 and thereby prevents the associated inflammatory signaling [132]. In another study, however, bromelain, ficin, and papain activated PAR2 and PAR4 by proteolytic cleavage, leading to increased intracellular calcium levels [133]. Thus, further studies are required to further clarify the modes of action of pineapple proteases.

### Cleavage-independent PAR activation by agonist peptides

Independent of proteolytic cleavage, PARs can be activated by synthetic soluble ligands corresponding with cleaved N-terminal sequences, or can be transactivated by cleavage-generated N-terminal regions of homo- or heterodimer partners.

Synthetic peptides that mimic the first six amino acids of tethered N-terminal ligands can act as agonist peptides that activate PARs in the absence of cleavage events [11, 18, 134]. Specific activation of PARs by a soluble agonist peptide was first shown for human PAR1 with the peptide SFLLRN [6, 18]. However, this peptide also activated PAR2 [135–137] and therefore various peptides were tested for specific PAR1 activation. Yet, PAR1 was the most specifically and efficiently activated by TFLLRN [138]. In addition to thrombin agonist peptides, other PAR1 agonist peptides have been identified. In particular, the peptide NPNDKYEPF reproduced the effects of aPC [28], and PRSFFLRN corresponds with the N-terminal peptide generated by MMP-1 [86]. SLIGKV corresponds with the trypsin cleaved N-terminal region of human PAR2. However, the corresponding rat N-terminus SLIGRL is a more specific and efficient PAR2 agonist in rodents and humans [136, 139], and only the synthetic peptide LIGRLO achieved this effect more efficiently than SLIGRL in humans [140]. The roles of ECL-2 in specific PAR activation have been shown using labeled PAR2 agonist peptides [141, 142]. Because the thrombin generated PAR3 peptide does not activate the G protein autonomously, no such agonist peptides have been identified to date [9, 143]. GYPGKF corresponds with the thrombin-cleaved human PAR4 and has weak activity as an agonist [144]. But replacement of the first amino acid glycine (G) with alanine (A) induced PAR4 by 10-fold. This peptide may be suitable as a platelet activator in humans and mice [145].

Several models of PAR–PAR interactions have been proposed and extensively studied based on PAR transactivation by agonist peptides [146]. When PAR1 is blocked on endothelial cells, however, thrombin, and not the PAR1-specific agonist peptide TFLLRN, induces signaling, reportedly by facilitating the heterodimerization of PAR1 and PAR2 [147]. Thrombin activation of the PAR1–PAR2 heterodimer leads to constitutive internalization and activation of β-arrestin by the PAR1 C-tail [146]. Accordingly, the required co-localization of PAR1 and PAR2 was shown in a human overexpression system, in mice studies of sepsis, and in PAR1–PAR2-driven cancer growth in a xenograft mouse model [148, 149]. In other studies, stable heterodimerization of human PAR1 and PAR4 was shown in platelet cells, and thrombin accelerated platelet activation under these conditions [150, 151]. Similar studies of mouse platelets showed efficient activation of platelets by thrombin in the presence of PAR3–PAR4 heterodimers [143]. Consistent with the thrombin-cleaved PAR3 peptide, which is not self-activating, PAR3 signaling was observed in the presence of PAR1 or PAR2 [22, 23, 34, 152]. Yet, heterodimerization influenced signal transduction and PAR membrane delivery due to enhanced glycosylation [153].

In addition to activation by heterodimerization, PARs interact with other receptors, such as ion channels, other G protein-coupled receptors (GPCRs), receptor tyrosine kinases (RTKs), receptor serine/threonine kinases (RSTKs), NOD-like receptors, and TLRs [154]. In particular, PAR2 initiated inflammatory signaling pathways, resulting in pain due to transactivation of the ion channels TRPV1 and TRPV4 in humans and mice [155–159]. Similar inflammatory effects follow transactivation of the RTKs EGFR and VEGFR by PAR2 and PAR4 [160–163]. Bacterial interactions with PARs suggest important roles of PARs in infectious disease. In agreement, TLRs recognize bacteria-derived molecules and contribute to innate immunity [164, 165]. Moreover, direct interactions of PAR2 with TLR3 and TLR4 were necessary for inflammatory responses to LPS in human cell lines and knockout mice and rats [166–171].

### PAR signaling

#### Activation pathways

PARs belong to a large family of GPCRs and induce multiple signaling pathways after coupling with heterodimeric G proteins. Activation of the Gα-subunit due to the exchange of a guanine from GDP to GTP results in dissociation of the Gβγ-dimer and activation of downstream pathways [172, 173].

**Fig. 4:**
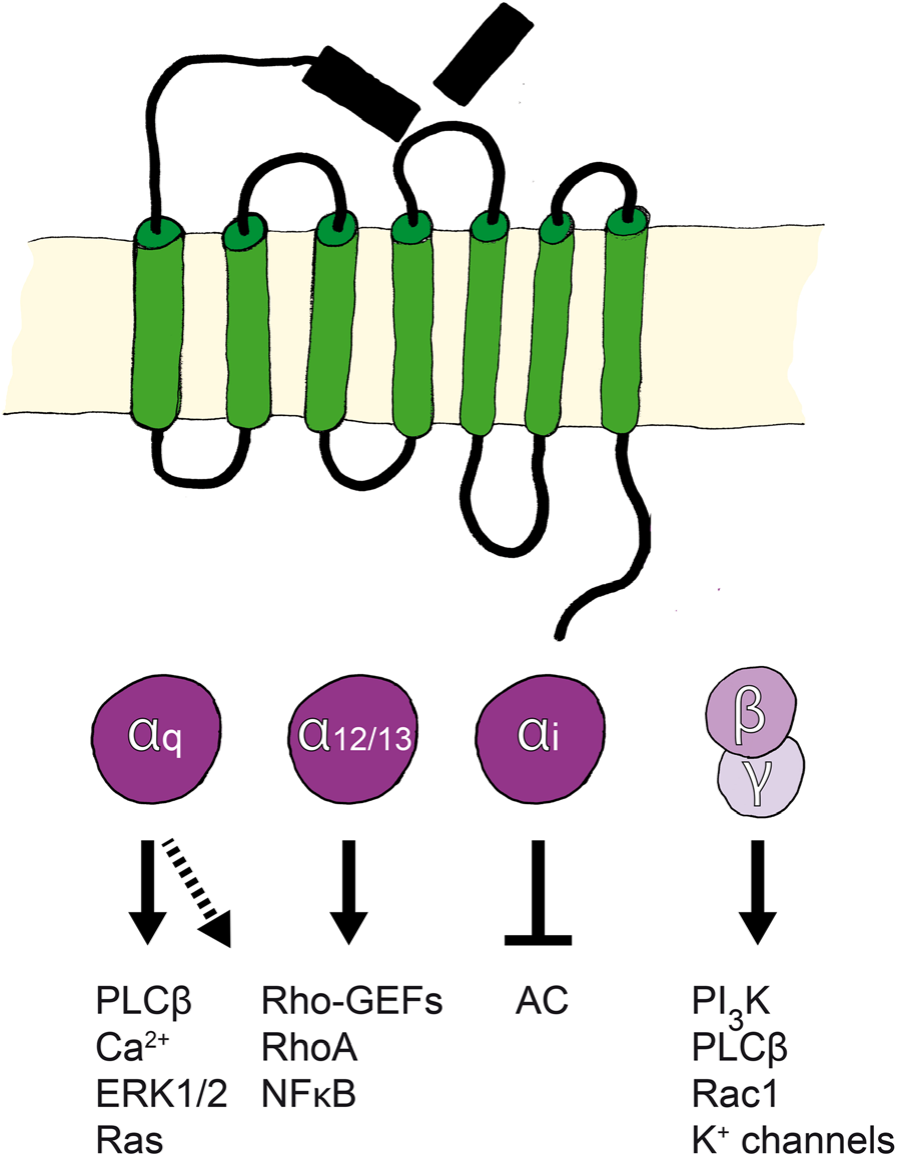
G protein-coupled signaling induced by PAR activation. Depending on the tethered ligand, activated PAR couples with G protein α-subtypes. Gαq activates phospholipase Cβ (PLCβ), which mobilizes calcium. This further activates MAPKs (ERK1/2) and induces Ras signaling. Primarily, Gα12/12 and Gaq activate the Rho pathway. Gαi inhibits the activation of adenylyl cyclase, which leads to reduced production of cAMP. In contrast, the βγ-subunit functions as a negative regulator when bound to the α-subunit. After receptor activation, subunits separate, and the βγ-subunit interacts with other proteins, thereby activating or inhibiting signaling

Following proteolytic cleavage or induction of agonist peptides, the engaged signaling pathways vary between tissues, cell lines, and the availability of co-receptors for transactivation. Depending on the ligand, specific α-subunits are activated, and these regulate subsequent cellular functions as summarized in Fig. 4. For example, thrombin-stimulated PAR1 activates the small GTPase protein RhoA via ERK1/2 kinases, but not via Rac1, whereas aPC-stimulated PAR1 induces Rac1 via Akt kinase, but not via RhoA [13, 174–176]. Moreover, in accordance with PAR1 cleavage sites, aPC prevents thrombin-induced RhoA signaling [16]. However, in contrast with thrombin-induced RhoA activation on platelets and endothelial cells, PAR1-agonist peptides and thrombin activated the inhibitory G protein G_i_ which leads to the inhibition of adenylyl cyclase in human fibroblasts [177, 178]. Other studies indicate that PAR2 activation is less tissue specific than PAR1 activation, and trypsin and VIIa cleaved PAR2 and activated G_αq_ and G_i_, resulting in calcium influx, MAPK activation, and inflammatory signaling [8, 179].

Signaling by tethered ligands can differ from that generated by corresponding soluble agonist peptides. For example, thrombin-cleaved PAR1 activated G_α12/13_ and G_αq_ and induced Rho and Ca^2+^ signaling, whereas the PAR1-agonist peptide activated only G_a12/13_ and downstream RhoA-dependent pathways that affected endothelial barrier permeability [180]. Similar observations of human platelets suggested that platelet activation followed coupling of thrombin-activated PAR1 with multiple heterotrimeric G protein subtypes, including G_α12/13_ and G_αq_ [181–183]. Moreover, trypsin and the PAR2-agonist peptide induced ERK1/2 signaling and inflammation by activating PAR2 [29, 180, 184–186]. β-arrestins also play major roles in PAR-induced signaling independently of G protein activation. For instance, aPC-activated PAR1 induces cytoprotective effects by recruiting β-arrestin in endothelial cells. Thus, aPC cleavage fails to protect β-arrestin deficient cells from the effects of thrombin [187, 188]. In addition, multiple studies show that activated PAR2 co-localizes with β-arrestin-1 and arrestin-2 and induces ERK1/2 signaling [77, 189–191].

#### Desensitization and termination

**Fig. 5:**
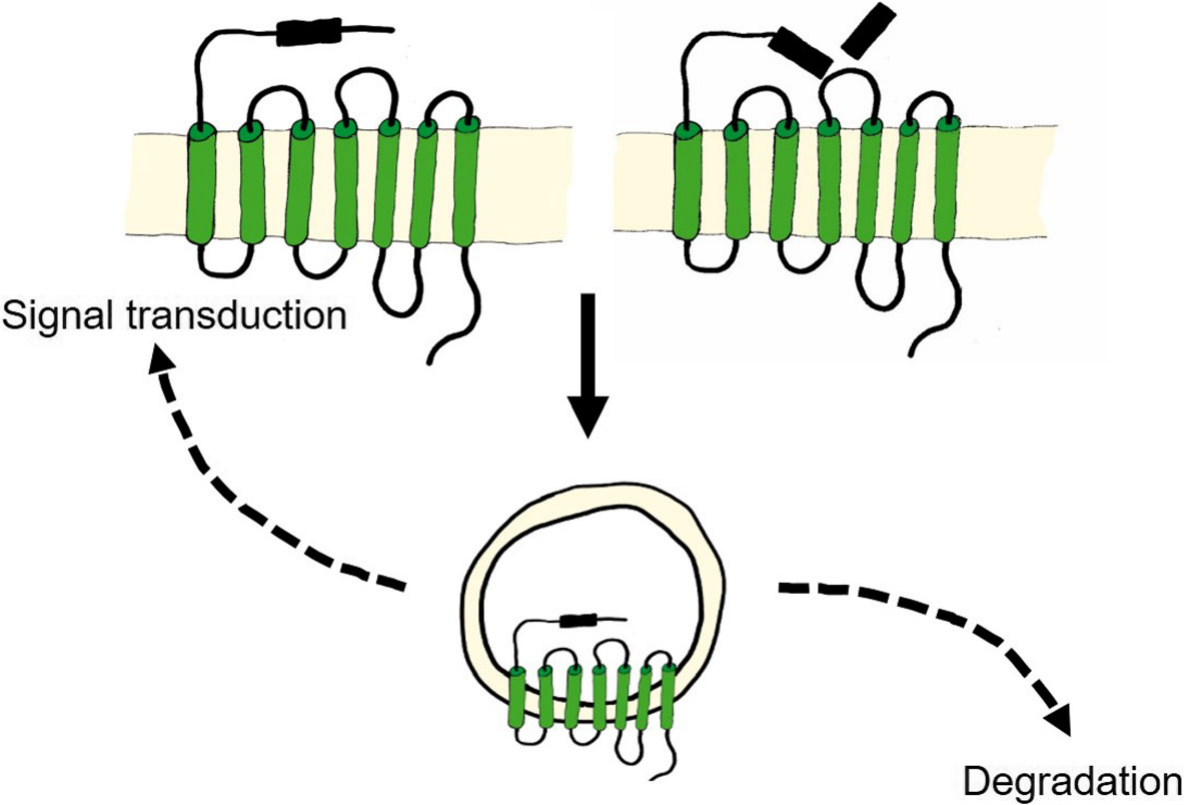
PAR trafficking. Activation-independent constitutive or agonist-induced internalization regulates PAR1 signaling

PAR activation is regulated by internalization and proteolytic desensitization, which limits the duration of signaling. For instance, PAR1 is constitutively internalized and recycled or agonist-induced internalized and degraded as described in [192, 193] and shown in the scheme of Fig. 5. As discussed above, some PAR-cleaving proteases abolish receptor responses by removing (shedding) or destroying the tethered ligands. For example, PAR1 is inactivated following cleavage by cathepsin G, and thrombin activation is hence prevented, allowing the formation of clotting under inflammatory conditions.

Depending upon proteolytic cleavage, PAR1 rapidly internalizes or accumulates on the cell surface [194, 195]. Activated PAR1 is internalized via clathrin- and dynamin-dependent mechanisms, and is sorted from early endosomes to lysosomes for degradation [196–199]. Although the mechanisms that terminate PAR1 signaling are not clearly understood, this process is known to involve phosphorylation, ubiquitination, and recruitment of β-arrestin [200–204]. In contrast with PAR1, activated PAR2 is not constitutively internalized [205]. Thus, to prevent persistent signaling upon activation, PAR2 is phosphorylated and ubiquitinated and then binds β-arrestin before being internalized and degraded [206–208]. Under these conditions, the activated and internalized PAR2 is not recycled and instead induces β-arrestin-dependent endosomal ERK1/2 signaling in the cytoplasm [189, 191, 209]. Thus, large cytoplasmic stores of newly generated PAR2 are required for rapid externalization and activation on cell membranes [210]. Although less is known about how PAR4 signaling is terminated, recent observations suggest that PAR4 internalization is independent of β-arrestin and slowly occurs via clathrin- and dynamin-dependent pathways [211]. In agreement, human platelets internalized PAR4 much slower than PAR1, and exhibited prolonged PAR4 signaling activity [212]. Moreover, growing evidence indicates that PAR–PAR heterodimerization is important for internalization, and that the underlying mechanisms include PAR2-dependent glycosylation of PAR4, thus affecting membrane transport [153]. Upon internalization, endosomal PAR4 dimerizes with the purinergic receptor P2Y12 and induces Akt signaling by recruiting β-arrestin within endosomes [213].

Depending on stimuli, PAR expression patterns are regulated by complex combinations of cell surface presentation, endocytosis, vesicle born or recycled (i.e., re-exocytosed) receptors, and trafficking modes that are linked to posttranslational modifications of PAR.

## Role of PARs in inflammation

With the current increases in the prevalence of inflammatory diseases, published in in vitro and in vivo studies of the roles of PARs in inflammation have become more numerous. These are reviewed below.

### Systemic inflammation and inflammatory cells in the cardiovascular system

PARs are critical for the interplay between clotting proteases of platelets, endothelial cells, and vascular smooth muscle cells that regulate hemostasis, vascular barrier function, vascular tone, vascular homeostasis, cell adhesion, and inflammatory responses [150]. The roles of PARs in these processes vary significantly between species. Specifically, whereas functional PAR1 and PAR4 are expressed in human platelets [214], PAR1, PAR3, and PAR4 have been found in guinea pig platelets [215]. Whereas mouse and rat platelets lack PAR1, they are activated at low concentrations of thrombin, which is recruited by PAR3 onto the surface of platelets and then efficiently activates PAR4 [4]. Due to interspecies differences in PAR expression, mouse and rat studies of PARs are difficult to translate to humans. PARs in endothelial cells contribute positive regulatory signals for endothelial adhesion molecules such as vascular cell adhesion molecule-1 (VCAM-1), intercellular adhesion molecule-1 (ICAM-1), and E-selectin [216, 217], all of which promote vascular barrier function. As a counterpart of intravascular cells, PAR4 induces leukocyte migration [75], and PAR2 expressed on macrophages promotes inflammatory modulators such as interleukin-8 (IL-8) [218]. These modes of signaling all contribute to a complex PAR-mediated interplay of endothelial cells that is orchestrated by intravascular cells and cytokine secretion. In addition, PARs, particularly PAR1, regulate vascular barrier function, and hence, extravasation of macromolecules such as complement proteins and antibodies. In addition, thrombin-mediated activation of PAR1 increases endothelial barrier permeability by activating mitogen-activated protein kinases (MAPKs) [219]. Although this effect is reversed by activated protein C (aPC)-mediated activation of PAR1 [28, 174, 175, 220]. Thrombin further promotes prostaglandin 2 (PGE2) secretion, and consequent endothelial barrier permeability [221]. Similarly, PAR1 activation increased vascular leakage in a murine model [222]. Inflammatory mediators, such as tumor necrosis factor alpha (TNFα), were shown to regulate the expression of endothelial PAR2, and the authors suggested that these data were indicative of barrier protective effects of PAR2 [223]. Several other studies show that PAR2 activation induces endothelium-dependent relaxation in blood vessels of mice and in arteries of rats [224–228]. In contrast, dual activities of PAR2 on blood vessels were reported in a study of rats [229]. In this line, thrombin-activated PAR1 induced the expression of vascular endothelial growth factor in smooth muscle cells [230], thus revealing the relationship between coagulation and vascular growth. Although the roles of PARs in the development of arteriosclerosis are yet to be elucidated, PAR2 and PAR4 were induced in human arteries under inflammatory conditions [223], suggesting important roles of PARs in vascular inflammation.

### Chronic inflammation of the gastrointestinal tract

In the gut lumen, human and bacterial proteases are both present at high concentrations. Similar to endothelial barriers, proteases regulate intestinal barrier permeability via PARs, all four of which are expressed by cells of the gastrointestinal tract [9, 224, 231, 232]. Trypsins and tryptases are prominent intestinal proteases, suggesting likely involvement of PAR2 as a major receptor of intestinal inflammation. In accordance, intestinal tight junctions are disrupted by PAR2-activating proteases, leading to inflammatory signaling in humans and rats [139, 206, 233, 234]. Although the roles of PARs in irritable bowel syndrome (IBS) and inflammatory bowel diseases remain unclear, roles of PARs in intestinal barrier function have been described. Specifically, PAR1 and PAR2 regulated permeability and chloride secretion, which are involved in diarrhea and constipation in IBS patients [234–236]. In addition, activated endosomal PAR2 caused persistent pain in a mouse model of IBS [209].

### Inflammatory diseases of the respiratory system

It has long been suggested that PARs are involved in the pathophysiology of respiratory disorders, reflecting observations of elevated levels of PAR-activating proteases, such as thrombin and tryptase, in bronchoalveolar lavage fluid from patients with pulmonary inflammation [237, 238]. In a sheep asthma model and in asthmatic patients, tryptase inhibitors reduced inflammation [239, 240], further indicating important roles of PAR2 in respiratory disease. These roles of PARs are also suggested by the prominence of a variety of non-mammalian PAR-activating proteases, such as those of house dust mites and cockroaches [120, 123, 124]. Expression of PAR1, PAR2, and PAR4 on bronchial epithelial and smooth muscle cells induced inflammatory signaling in multiple studies [55, 121, 241–245]. PAR2 is also upregulated in epithelial cells of patients with asthma and chronic obstructive pulmonary syndrome (COPD) [246, 247]. Whether PAR2 activation results in bronchoconstriction or dilatation remains controversial, in part owing to interspecies differences and tissue dependencies [242, 248, 249]. In humans, however, PAR1-agonist peptides with thrombin, and a PAR2-agonist peptide with trypsin and tryptase, induced bronchoconstriction by inducing Ca^2+^ signaling in airway smooth muscle cells [241, 244]. Moreover, the long-term activation of PAR1 and PAR2 led to pulmonary fibrosis in mice models [250].

### Inflammatory skin diseases

High concentrations of exogenous proteases are present on the skin of various species, and these may activate PARs to regulate epidermal permeability and barrier function [251]. Indeed, epidermal inflammation has been linked to PAR1 and PAR2 activation in keratinocytes, which comprise the epidermal barrier with sub-epidermal skin fibroblasts [179, 252, 253]. Subsequent release of IL-8, IL-6, and granulocyte macrophage colony-stimulating factor (GM-CSF) was also observed previously [254], potentially involving NFκB activation [255]. In addition, the inflammatory roles of PAR2 have been demonstrated in mice models of atopic dermatitis due to elevated tryptase and PAR2 expression levels [256, 257]. Similar to studies in mouse models, PAR2 was upregulated in patients with atopic dermatitis, and PAR2 agonists increased itch, causing irresponsiveness of sensory nerves to therapy with antihistamines [258].

### Rheumatic disease

“Rheumatic disease” is a common term for autoimmune diseases that affect joints, bones, and muscles. Although rheumatic disorders are numerous, some of the common underlying symptoms include chronic joint inflammation, stiffness, and pain [259]. Currently, PAR2 is the only PAR that has been associated with the development of rheumatic diseases [260]. Direct roles of PAR2 in rheumatic diseases were first indicated in 2003 in a mouse study by Ferrell et al. [261]. In their study, a PAR2-agonist peptide induced strong inflammatory effects in wt mice, causing joint swelling and synovial hyperemia, whereas joint swelling was absent in PAR2 deficient mice [261]. Similarly, in patients with rheumatoid arthritis, PAR2 is upregulated in inflamed tissues [262]. Further increases in PAR2 expression were noted in monocytes, and the PAR2-agonist peptide upregulated IL-6. In contrast, PAR2 expression was decreased after treatments with antirheumatic drugs [263], further supporting the role of PAR2 in rheumatic disease.

## PAR modulators as targets for therapy

**Fig. 6:**
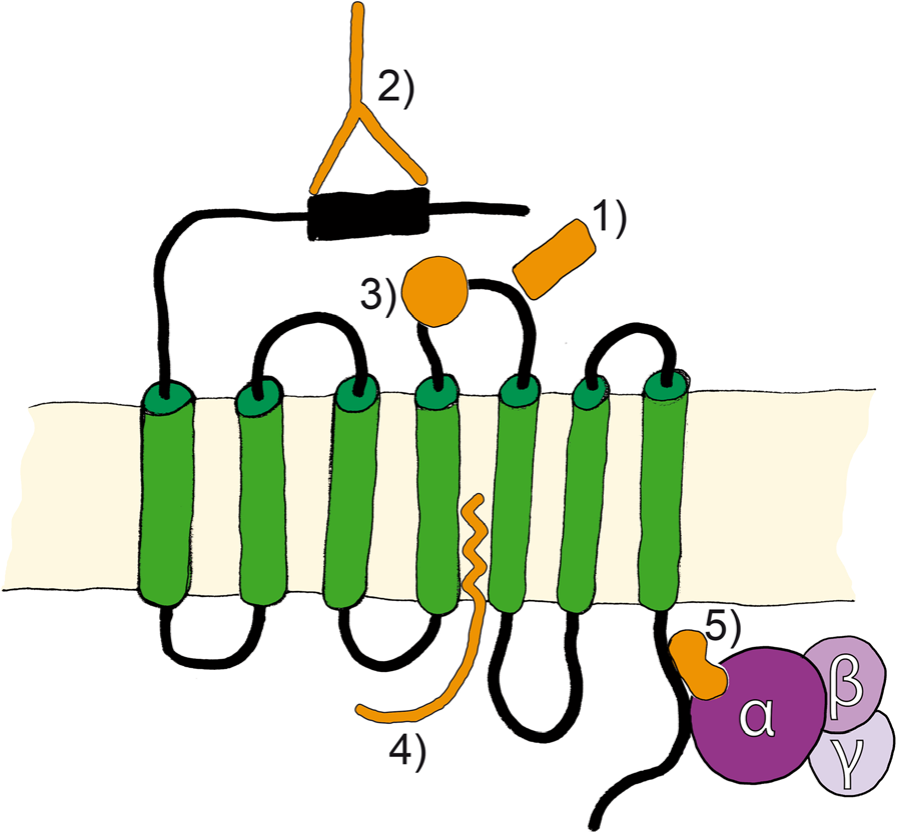
PAR modulators. Pharmacological substances, such as 1) peptides and peptidomimetics, 2) blocking antibodies, 3) small molecules, 4) pepducins, and 5) parmodulins are used as therapeutic agents that affect PAR activities

The complexity of PAR regulation is indicated by the culmination of specific proteolytic cleavage modes (inactivating or activating), protease inhibitors, and cofactors, and with the effects of PAR glycosylation and dimerization (Fig. 1). In this section we discuss classes of agonists and antagonists that have been tested as PAR modulators for use as therapeutic agents as summarized in Fig. 6 and Tables 5, 6 and 7.

**Table 5:** PAR1 signaling modulators

Class	Agonist/ Antagonist	Name	Receptor/Cell/Tissue type	Cellular response
Peptide	Agonist	SFLLRN/−NH2	Human	Induces platelet activation [6, 138, 265, 278, 279]
		TFLLRN/−NH2	Human	Induces platelet activation, enhances endothelial barrier permeability [137, 138, 265]
		NPNDKYEPF/−NH2	Human	Induces cytoprotective signaling [28, 187]
		PRSFLLRN/−NH2	Human	Induces platelet activation [86]
			Human	Induces ERK1/2 activation [280]
	Antagonist	YFLLRN	Human	Compets with thrombin and PAR1-AP and prevents platelet activation [278, 279]
Peptidomimetic	Antagonist	RWJ-56110	Human	Blunts thrombin and PAR1-AP effects on platelets and vascular endothelial cells [264, 281, 282]
			Human	Blocks MMP-1 activaiton in SMCs [87]
		RWJ-58259	Guinea pig	Blocks thrombin and PAR1-AP platelet activation [215, 283]
			Rat	Blocks thrombin induced calcium release in AoSMC Inhibits intimal thickening [111, 215, 264, 273]
			Mouse	Prevents destruction of intestinal barrier [62, 284]
Non-peptide small molecule	Antagonist	FR17113	Human	Blocks PAR1-AP induced platelet activation [285, 286]
			Human	Inhibits thrombin and PAR1-AP induced ERK1/2 activation [287]
		ER129614–06	Human	Blocks thrombin and PAR1-AP induced platelet activation [288]
			Guinea pig	Shows antithrombotic effects [289]
		F16357, F16618	Human	Blocks PAR1-AP induced platelet activation [290]
			Rat	Shows antithrombotic effects [291]
		SCH79797	Human	Blocks thrombin and PAR1-AP induced calcium release and platelet activation [292]
			Human, Mouse	Induces NETs formation and increases bacterial killing capacity [293]
		SCH203009	Human	Blocks thrombin and PAR1-AP induced platelet activation [292]
		SCH530348 (vorapaxar)	Human, Monkey	Blocks thrombin and PAR1-AP induced platelet activation [266]
		E5555 (atopaxar)	Human	Blocks thrombin and PAR1-AP induced platelet activation and inhibits thrombus formation [267]
			Guinea pig	Bleeding time not affected [267, 294]
		Q94	Human	Blocks thrombin induced calcium release [295]
			Mouse	Blocks thrombin induced ERK1/2 activation [296]
Pepducin	Antagonist	P1pal-12	Human	Blocks thrombin induced platelet activation [268]
			Human	Blocks platelet activation [86]
			Human	Blocks MMP-1 induced endothelial damage [297]
			Mouse	Reduces lung vascular damage and sepsis lethality [297, 298]
		P1-pal7 (PZ-128)	Human	Blocks MMP-1 induced Akt signaling in cancer cells [150]
			Human	Blocks platelet activation [86]
			Mouse	Inhibits tumor growth [280]
			Guinea pig	Prevents from systemic platelet activation [86]
Parmodulin	Antagonist	ML161 (Parmodulin-2)	Human	Blocks thrombin and PAR1-AP induced platelet activation [299]
			Human	Blocks thrombin induced inflammatory signaling on endothelial cells [269]
			Mouse	Blocks thrombus formation [300]
Antibiotic	Antagonist	Doxycycline	Human	Inhibits thrombin induced cancer cell migration [301, 302]
			Human	Blocks MMP-1 cleavage [303]
Antibody	Antagonist	ATAP-2 WEDE	Human	Blocks thrombin cleavage of PAR1 and thrombin induced calcium release [147]

**Table 6:** PAR2 signaling modulators

Class	Agonist/ Antagonist	Name	Receptor/Cell/Tissue type	Cellular response
Peptide	Agonist	SLIGRL/−NH2	Human, Rat	Induces calcium release [2, 8, 136, 139]
		SLIGKV/−NH2	Human	Induces calcium release [136]
		2f-LIGRLO/−NH2	Human, Rat	Induces calcium release [140]
	Antagonist	FSLLRY-NH2	Human	Blocks trypsin, not SLIGRL activation, reduces proinflammatory IL-8 and TNFα [82]
			Rat	Inhibits neuropathic pain [304]
		LSIGRL-NH2	Human	Blocks trypsin, not SLIGRL induced calcium release [305]
Peptidomimetic	Antagonist	K14585, K12940	Human	Reduces SLIGKV induced calcium release [306]
			Human	Inhibits SLIGRL induced NFkB activation [307]
		C391a	Human, Mouse	Blocks calcium release and MAPK activation [308]
Non-peptide small molecule	Agonist	GB110	Human	Induces calcium release [309]
		AC-5541, AC-264613	Human	Induces calcium release [310]
			Rat	Induces edema and hyperalgesia [310]
	Antagonist	ENMD-1068	Human	Blocks p.acnes induced calcium release and induction of IL-1a, IL-8 and TNFα [92]
			Human	Inhibited FVIIa induced cancer cell migration [311]
			Mouse	Reduces joint inflammation [260]
			Mouse	Blocks calcium release and reduces liver fibrosis [312]
		GB83	Human	Inhibits trypsin and PAR2-AP calcium release [313]
		GB88	Human	Blocks PAR2 induced calcium release [309]
			Rat	Reduces acute paw edema, inhibits PAR2-AP induced inflammation [309, 314]
		AZ8838 AZ3451	Human	Blocks PAR2-AP induced calcium release and β-arrestin recruitment [315]
Pepducin	Antagonist	P2pal-18S	Human	Blocks PAR2 induced calcium release [316]
			Mouse	Decreases risk for developing severe biliary pancreatitis [317]
		P2pal-14GQ	Human	Blocks PAR2 induced calcium release [316]
Antibiotic	Antagonist	Tetracyclines (Tetracycline, Doxycycline, Minocycline)	Human	Inhibits SLIGRL induced IL-8 release [318]
			Mouse	Topical application of tetracycline decreases PAR2 induced skin inflammation [319]
			Rat	Subantimicrobial doses of doxycycline inhibit PAR2 induced inflammation [320]
Antibody		SAM-11	Mouse	Reduces joint inflammation [260]
			Mouse	Prevents allergic inflammation [124]
		B5	Mouse	Reduces joint inflammation [260]
			Mouse	Inhibits allergic airway inflammation [124]
		MAB3949	Human	Blocks trypsin induced PAR2 activation [315]

**Table 7:** PAR4 signaling modulators

Class	Agonist/ Antagonist	Name	Receptor/Cell/Tissue type	Cellular response
Peptide	Agonist	GYPGQV/−NH2	Human, Rat	Induces platelet activation [144]
		GYPGKF/−NH2	Human, Rat	Induces platelet activation [144]
		AYPGKF/−NH2	Human, Mouse	Induces platelet activation [145]
Peptidomimetic	Antagonist	tc-YGPKF	Rat	Blocks thrombin and PAR4-AP induced platelets aggregation [321]
Non-peptide small molecule	Antagonist	YD-3	Human	Blocks thrombin induced platelet activation [282, 322–325]
			Mouse, Rat, Rabbit	Blocks thrombin and PAR4-AP induced platelets activation [323–325]
		ML-354	Human	Blocks PAR4-AP induced platelet activation [326–328]
		BMS-986120	Human	Blocks PAR4-AP induced calcium release and platelet activation [329]
			Human	Blocks thrombus formation at high shear stress [277]
			Monkey	Blocks platelet activation [329]
Pepducin	Antagonist	P4pal-10	Human, Mouse	Blocks thrombin and PAR4-AP induced platelet activation [268]
			Rat	Blocks thrombin and PAR4-AP induced platelets activation [330]
		P4pal-i1	Human	Blocks PAR4 induced platelets activation [150]

Peptide agonists and antagonists are short synthetic peptides that mimick the PAR-tethered ligand that is liberated by proteolytic cleavage, as described above. These peptides either induce signal transduction or prevent cleavage-dependent signaling following PAR rapid internalization, and some C- or N-terminal modifications of soluble ligand sequences have resulted in increased activation efficiency [18]. Peptidomimetic antagonists are small protein-like chains that mimick the tethered ligands of PARs, and were recently used as PAR modulators for the first time [264].Soon after PARs were discovered, PAR1 blocking antibodies were reported [265], and these blocked protease binding and or the cleavage site of the receptor. Non-peptide small molecules, such as the PAR1 antagonists vorapaxar [266] and atopaxar [267], also interact with PARs, mainly via ECL2.Only two classes of intracellular PAR antagonists have been developed to date. Pepducins are cell penetrating palmitoylated peptides that were derived from the intracellular loop of PAR, and these interfere with G protein binding [268]. Parmodulins, in contrast, are small molecules that bind PARs at the G protein binding pocket of the C-tail to compete with G_αq_ subunits, but not with other G_α_ subunits [269].

### Examination of agonists and antagonists in vitro and in preclinical studies (Tables 5, 6 and 7)

### Clinical studies

Despite the importance of PARs in various pathophysiological conditions, few PAR modulating tools have been tested in clinical studies, and even fewer have been established for treatment. Since the identification of PAR1 as a platelet thrombin receptor, an abundance of research has been conducted to identify PAR1 antagonists that can block platelet activation and prevent thrombotic cardiovascular events. The first clinically approved PAR1 antagonist was the small-molecule antagonist vorapaxar [266]. Phase II clinical trials of this agent showed reduced risks for myocardial infarction in patients treated with vorapaxar in combination with standard antiplatelet therapy. Moreover, the risks of bleeding complications were not significantly increased [270]. Subsequently, two large-scale phase III multicenter, randomized, double-blind, placebo-controlled studies of vorapaxar (ZONTIVITY, SCH530348) were performed. In the Thrombin Receptor Antagonist in Secondary Prevention of Atherothrombotic Ischemic Events–Thrombolysis in Myocardial Infarction 50 (TRA 2°P-TIMI 50; details at www.ClinicalTrials.gov; NCT00526474) study, the rate of cardiovascular events at the second efficacy endpoint were significantly reduced by vorapaxar in combination with standard antiplatelet therapy [271]. Furthermore, in the Thrombin Receptor Antagonist for Clinical Event Reduction in Acute Coronary Syndrome (TRACER; details at www.ClinicalTrials.gov; NCT00527943) study, vorapaxar reduced the hazard of first myocardial infarction of any type in patients who were treated within 24 h of having symptoms of a cardiovascular event. However, in the TRACER study, vorapaxar failed to prevent secondary ischemic events [272]. Because vorapaxar increased bleeding complications in the clinical setting, the alternative PAR1 antagonist atopaxar (E5555) [267] was tested in a phase II clinical trial called (Lessons From Antagonizing the Cellular Effects of Thrombin-Acute Coronary Syndromes (LANCELOT-ACS; details at www.ClinicalTrials.gov; NCT00548587) study [273]. Atopaxar inhibited platelet aggregation in ACS patients in a dose-dependent manner, and caused no side effects of abnormal platelet activation, such as bleeding [274, 275]. Yet, patients receiving atopaxar had dose-dependent increases in liver abnormalities [273].

To prevent the bleeding problems that arise from treatments with PAR1 antagonists, a new class of PAR1 antagonist was designed, and the member pepducin PZ-128 (P1-pal7) was tested in a phase I trial [276]. This study showed no reduction in platelet aggregation, but the platelet blocking effect of PZ-128 was reversible ex vivo in the presence of saturating concentrations of the PAR1 agonist peptide SFLLRN. Based on these promising findings, the new PAR1 blocking agent PZ-128 was considered in the coronary artery disease study Thrombin Receptor Inhibitory Pepducin-Percutaneous Coronary Intervention (TRIP-PCI). Data from this phase II trial are not yet available (details at www.ClinicalTrials.gov; NCT02561000).

As an alternative to PAR1 targeted antithrombotic drugs, the PAR4 small-peptide antagonist BMS-986120 reduced reversible thrombus formation ex vivo in a phase I trial [277]. Consequently, this promising anticoagulant PAR4 antagonist is currently being compared with a standard anticoagulant drug in a phase II study of stroke recurrence (details at www.ClinicalTrials.gov; NCT02671461).

## Conclusion

Since the identification of PARs in the 1990s, studies of the complex mechanisms of PAR activation have been abundant, and these have clarified the roles of PARs in inflammatory disease. Various mammalian and non-mammalian proteases have also been recognized as PAR-mediated regulators of physiological and pathophysiological processes. Despite the development of various PAR modulators, few have been approved for therapeutic use. Obstacles to this therapeutic strategy include species differences in PAR expression and limited bioavailability of modulators in vivo and in clinical studies. Further research is needed to identify specific and efficient anti-inflammatory PAR modulators.

## Abbreviations

AC: Adenylyl Cyclase
AKT: Protein kinase B
aPC: Activated Protein C
COPD: Chronic Obstructive Pulmonary Syndrome
COX: Cyclooxygenase
ECL: Extracellular Loop
EGFR: Epidermal Growth Factor Receptor
EPCR: Endothelial Protein C Receptor
ERK: Extracellular Signal-regulated Kinase
F2R: Coagulation Factor 2 Receptor
F2RL1: Coagulation Factor 2 Receptor-Like 1
F2RL2: Coagulation Factor 2 Receptor-Like 2
F2RL3: Coagulation Factor 2 Receptor-Like 3
FVIIa: Activated Coagulation Factor VII
FXa: Activated Coagulation Factor X
GM-CSF: Granulocyte Macrophage Colony-stimulating Factor
GPCR: G Protein-Coupled Receptor
IBD: Inflammatory Bowel Disease
IBS: Irritable Bowel Syndrome
ICAM: Intercellular Adhesion Molecule
IL: Interleukin
LepA: *Pseudomonas aeruginosa*-Derived Large Extracellular Protease
LPS: Lipopolysaccharides
MAPK: Mitogen-Activated Protein Kinase
MMP: Matrix Metalloprotease
NFκB: Nuclear Factor kappa B
PAR: Protease-Activated Receptor
penC: Penicillium citrinum-Derived Alkaline Serine Protease C
PGE2: Prostaglandin E2
PI3K: Phosphatidylinositol-3-Kinase
PLCβ: Phospholipase C beta
Rac1: Ras-Related C3 Botulinum Toxin Substrate 1
RhoA: Ras homolog gene family, member A
RSTK: Receptor Serine/Threonine Kinase
RTK: Receptor Tyrosine Kinase
SpeB: Streptococcal Pyrogenic Exotoxin B
TF: Tissue Factor
TLR: Toll-Like Receptor
TM: Thrombomodulin
TRPV: Transient Receptor Potential Channels Vanilloid Subtype
VCAM: Vascular Cell Adhesion Molecule
VEGF: Vascular Endothelial Growth Factor
VEGFR: Vascular Endothelial Growth Factor Receptor
