# Correction to: protease-activated receptors (PARs): mechanisms of action and potential therapeutic modulators in PAR-driven inflammatory diseases

**DOI:** 10.1186/s12959-019-0212-x

**Published:** 2019-11-06

**Authors:** Dorothea M. Heuberger, Reto A. Schuepbach

**Affiliations:** 1 Institute of Intensive Care Medicine, University Hospital Zurich, University of Zurich, Zurich, Switzerland; 2 Surgical Research Division, University Hospital Zurich, University of Zurich, Zurich, Switzerland

**Correction to: Thrombosis Journal (2019) 17:4.**

**http://doi.org/10.1186/s12959-019-0194-8**

Following the publication of this article [1], the authors reported an incorrect citation in the following sentence in the “Metalloproteases” sub-section:

“Similarly, MMP-2 cleaves human PAR1 and enhances platelet activation [91], and MMP-3, MMP-8, and MMP-9 were shown to induce platelet activation via PAR1 [92].”

The correct citation for reference 92 in this sentence is: Lee EU, Woo MS, Moon GP, Baek MC, Choi IY, Kim WK, Junn E and Kim HS. α-Synuclein Activates Microglia by Inducing the Expressions of Matrix Metalloproteinases and the Subsequent Activation of Protease-Activated Receptor-1. J Immunol. 2010, ji_0903480.

For further clarification, this sentence should read as follows:

“Similarly, MMP-2 cleaves human PAR1 and enhances platelet activation [91], and MMP-3, MMP-8, and MMP-9 were shown to cleave and activate PAR1 peptide at thrombin cleavage site R41 [92].”
